# Exploring key challenges for healthcare providers and stakeholders in delivering adolescent sexual and reproductive health services and information during the COVID-19 pandemic in Malawi, Zambia and Zimbabwe: a qualitative study

**DOI:** 10.1186/s12913-024-11873-5

**Published:** 2024-12-04

**Authors:** Margarate Nzala Munakampe, Tulani Francis L. Matenga, Mwimba Chewe, Anise Gold-Watts, Reza Lahidji

**Affiliations:** 1 Department of Health Policy and Management, School of Public Health, University of Zambia, Lusaka, Zambia; 2 Yakini Health Research Institute, Lusaka, Zambia; 3 Department of Health Promotion and Education, School of Public Health, University of Zambia, Lusaka, Zambia; 4 KPMG International Development Advisory Services, Oslo, Norway

**Keywords:** COVID-19, Adolescents, SRHR, Malawi, Zambia, Zimbabwe, HIV testing, Teenage pregnancy

## Abstract

**Introduction:**

This study aimed to explore the direct and indirect influences of COVID-19-related restrictions on adolescents and young people’s (AYP’s) sexual and reproductive health and rights (SRHR) in Malawi, Zambia, and Zimbabwe, with a focus on teenage pregnancy and access to and utilization of HIV testing and counselling services.

**Methods:**

Thirty-four purposively sampled interviews that comprised of selected representatives of organizations involved in activities aimed at addressing adolescent sexual and reproductive health and rights (ASRHR), teenage pregnancies, and HIV testing were conducted in Malawi, Zambia and Zimbabwe. In Zambia, the study conducted an additional four group discussions with adolescents and young people. Adolescents and young people were asked to describe their experience and perceptions of the impact of COVID-19 on their SRHR. Thematic analysis was used to analyze the data.

**Results:**

Priority shifts resulted in the focus of service provision to the COVID-19 response. This led to shortages of already insufficient human resources due to infection and/or isolation, supply chain disruptions leading to shortages of important sexual and reproductive health (SRH)-related commodities and supplies, compromised quality of services such as counselling for HIV and overall limited AYP’s access to SRH information. Suggestions for interventions to improve SRH services include the need for a disaster preparedness strategy, increased funding for ASRHR, the use of community health workers and community-based ASRHR strategies, and the use of technology and social media platforms such as mHealth.

**Conclusion:**

Disruption of SRH services for adolescents and young people due to pandemic related-restrictions, and diversion of resources/funding has had a ripple effect that may have long-term consequences for adolescents and young people throughout the East and Southern African region. This calls for further investment in adolescents and young people’s access to SRHR services as progress made may have been deterred.

**Supplementary Information:**

The online version contains supplementary material available at 10.1186/s12913-024-11873-5.

## Introduction

According to the World Health Organization (WHO), nearly 12 million adolescent girls and young women aged 15–19 years and nearly a million under 15 years old give birth every year [1]. The majority of these births occur in sub-Saharan Africa with the highest adolescent fertility rate over the period 2015–2020 at 102.8 births per 1000 person-years, over 2 times higher than the global average (44 per 1000) [2]. The overall prevalence of teenage pregnancy- adolescent pregnancy in a female under 20 years in Africa is 18.8%, with slightly greater proportions in East Africa (21.5%) and Southern Africa (20.4%) [3], indicative of poor access to sexual reproductive health (SRH) information and services. In East and Southern Africa, SRH issues are highly contested, especially for adolescents and young people (AYP), and this has perpetuated service accessibility and utilization challenges. Young people often face major sexual and reproductive health challenges, including a lack of access to services and high levels of child marriage, early childbearing, unintended pregnancy, unsafe abortion, gender-based violence (GBV), and exposure to sexually transmitted infections (STIs), including HIV [4].

Reduced access to services often stems from health worker bias or a lack of willingness to provide services, financial constraints, or the lack of knowledge about sexual health needs [5] and the vulnerability associated with these. Adolescent pregnancies usually occur in marginalized communities, driven by a lack of education access and income-earning opportunities [6]. Furthermore, gaps in access to SRH information and services leave AYP more susceptible to ‘illegal’ or ‘unsafe’ options- such as unsafe abortion [7–9]. Other negative consequences include an unmet need for contraception, harmful gender inequalities, high sexual gender-based violence (SGBV) rates, and drug and alcohol abuse [6, 10, 11].

Although strides in realizing SRH rights for young people have been made, the onset of the COVID-19 pandemic has left overwhelming economic, social, as well as health effects in countries around the world, leaving low- and middle-income countries (LMICs) vulnerable to negative effects amid their already fragile systems. The pandemic at its peak resulted in about 5 million deaths worldwide, battered health systems, and damaged livelihoods through a reduction in the workforce and increased unemployment [12]. Evidence suggests that the disruptions caused by the pandemic have led to negative consequences for AYP due to their prior vulnerability [13, 14]. Adolescence is a critical period that is often characterized by physical and psychosocial developmental changes [15]. It is a time when individuals develop their place in society (notably by developing social relations), and their sense of self, and become more independent and therefore particularly vulnerable to the disruptions that COVID-19 has caused. The pandemic and its accompanying control measures disrupted many routine activities, altering the well-being of young people, and therefore had a disproportionate impact on AYP [16, 17].

The onset of the COVID-19 pandemic led to global, regional, and local measures to reduce disease transmission, including bans on public gatherings, compulsory stay-at-home restrictions (e.g., lockdowns), mandatory closures of nonessential businesses, facemask ordinances to name a few [18]. Although AYP may have appeared to be less at risk for severe COVID-19 symptoms and effects, the pandemic significantly had an impact on their health and well-being too [19]. In April 2020, UNESCO warned that “the potential for increased dropout rates which will disproportionately affect adolescent girls, further entrench gender gaps in education and lead to increased risk of sexual exploitation, early pregnancy and early and forced marriage” would rise as a result of the pandemic and its associated measures [20]. The COVID-19 pandemic and control measures deterred most of the strides in increasing access to and utilization of SRH services by AYP [21]. Numerous studies documented restrictions in access to contraceptives; HIV services – including medical male circumcision, sexually transmitted infections (STI) screening and management services, and safe abortion/post-abortion care, due to the collective effort of health facilities to prioritize COVID-19 response over these services [22–24]. AYP faced a reduction in the availability of sexual and reproductive health rights (SRHR) information and services, youth-friendly health services, and particularly, access to contraception and HIV testing and counselling [13, 16, 21].

At the beginning of the pandemic, researchers and experts, based on lessons learned from the Ebola epidemic of 2014 as a reference point, widely speculated that COVID-19 may have a detrimental influence on SRH service needs, access, and use in low- and middle- income countries. Additionally, while recent studies in other countries documented COVID-19’s impact on women’s SRHR experiences, with a particular focus on fertility, childbearing preferences, and contraceptive access [25], a deeper understanding of the impact of a pandemic on the delivery of SRH, HIV and GBV services to AYP in Zambia, Zimbabwe and Malawi remains necessary [26]. This study, therefore, aimed to explore the direct and indirect influences of COVID-19-related restrictions on adolescents and young people’s SRHR in Malawi, Zambia, and Zimbabwe. Specifically, the study focuses on teenage pregnancy as well as access to and utilization of HIV testing and counselling services to highlight the prevailing situation of SRHR among young people in the three countries. Further, the study aims to inform timely and appropriate remedial actions and service provision in pandemic situations and to identify strategies to prioritize SRHR during subsequent global pandemics.

## Methods

### Study design

We adopted a qualitative case study of access to SRH services during the COVID-19 pandemic in Malawi, Zambia and Zimbabwe over the period 2020–2021. This was done as part of an in-depth, mixed-methods design combining a scoping review; secondary analysis of national-level health information systems data; qualitative focus group discussions (FGDs); and key informant interviews (KIIs). We conducted an explanatory sequential mixed-methods design where secondary quantitative data was first collected. Perceptions of AYP, service providers, and other key stakeholders were then collected through KIIs and FGDs who included adolescents and youth-led/serving organizations, community-based organizations, relevant NGOs, relevant units/directorates in the line ministries (e.g., Health, Education, and Youth), and other strategic partners and stakeholders. In Zambia, AYP were included in the study and asked to describe their experience/perceptions of the impact of COVID-19 on AYP’s SRHR. A case study design was chosen for this research due to its ability to provide in-depth and contextual understanding of the the topic [27].

### Study context: Malawi, Zambia, and Zimbabwe

Malawi, Zambia, and Zimbabwe have young population age structures with more than 40% of the population aged below 15 years and less than half of sexually active unmarried women aged 20–24 currently using modern contraceptives. All three countries are conservative concerning the sexual practices of unmarried adolescents, reflected in the reluctance of parents and healthcare providers to support adolescent contraceptive use. In addition to limited services, SRH use is constrained in all three countries by concerns including confidentiality, providers’ biases, and low levels of knowledge. Additionally, most AYP in sub–Saharan Africa live in families and communities struggling with persistently poor socio-economic conditions, inequities, and socially constructed gender roles that undermine their health [28]. The well-being and prospects of AYP are further undermined by vulnerability to HIV, STIs, unintended pregnancy and unsafe abortions. A multiplicity of structural and sociocultural factors contribute to these poor adolescent SRH outcomes [29].

### Selection of participants

In this study, a purposive sampling strategy was employed to select representatives of organizations to participate in discussions across the three country context capitol cities, Lilongwe, Lusaka and Harare. Participants were selected based on their organization’s involvement in activities aimed at addressing ASRHR, teenage pregnancies, and HIV testing. KIIs were conducted with target providers, line ministries, and other partners. The research team had no prior relationships with the study participants hence a snowball approach was also used to select participants that had played a role in addressing adolescent health issues at the frontlines as well as at the managerial level, in consultation with key stakeholders involved in ASRHR issues. The research team then followed up by inviting the participants for an interview on activities aimed at addressing ASRHR, teenage pregnancies, and HIV testing during the COVID-19 pandemic. Data saturation was reached with a total of 34 KIIs and four FGDs across the three countries. AYP in Zambia were identified through a local community-based NGO that works with AYP in Lusaka, through sports (Table 1).

**Table 1:** Participant Characteristics

	Malawi		Zambia		Zimbabwe	
Stakeholder Organization	Gender	Number of participants	Gender	Number of participants	Gender	Number of participants
Government Ministries, Departments, and National Councils						
Ministry of Youth	M	1	F	1		
Ministry of Education/District Education Board	F	1	M	1		
Social Welfare			F	1		
Victim Support Unit (Police)			M	1		
Teachers (Guidance & Counselling)			F	1		
In-service Training Centre			F	1		
Ministry of Health	M	1	F	1		
National AIDS Council					5(M)/1 (F)	6
**Donors**						
Bilateral Agency	F	1				
International NGO	F	1				
International NGO	M	1				
**Local NGOs (Working with adolescents)**						
Local NGO 1			M	1		
Local NGO 2	F	1	F	1		
Local NGO 3	F	1				
Local NGO 4	M	1			M	1
Local NGO 5	M	1	F	1	M	1
Local NGO 6					F	1
Local NGO 7					M(4)/F(3)	8
Local NGO 8					M	1
Local NGO 9					M	1
Local NGO 10					M	1
Local NGO 11					F	1
**Clinics (Public)**						
ASRHR Section	M	1	F	1		
Government Hospitals			F	1		
**Clinic (Private / NGOs)**						
NGO 1	M	1				
NGO 2	F	1				
NGO 3	M	1				
**Religious Leaders**						
Religious Leader			M	1		
**AYP Focus Group Discussions**						
Girls (15–18 Years)			F	6		
Boys (15–18 Years)			M	6		
**ASRHR Volunteers**						
Peer Counsellor			M	1		

### Data collection

Data was collected between July 2022 and October 2022. Participants were offered the choice of an interview either in person or via Zoom. Data collection was carried out respecting the COVID-19 preventive measures of each targeted country when conducting in-person interviews. Phone call interviews were also done, including virtual discussions on platforms such as Microsoft Teams, and/or zoom. An interview guide was developed for primary data collection and each interview lasted between 30 and 60 min. Consultative sessions were conducted using FGDs, where each FGD had between 6 and 8 participants and lasted 60 to 190 min. The FGDs allowed for further probing of information on specific issues from the perspective of AYP. MNM, TFLM and AGW conducted the interviews and focus group discussions, assisted by local in-country trained qualitative research assistants.

### Data analysis

When available and in cases where the participants agreed to be audio-recorded, the recorded discussions were transcribed verbatim with analysis initiated alongside the data collection. In total, ten interview participants were not audio-recorded, therefore detailed notes substituted verbatim transcription. The analysis process began with listening to the audio recordings against reading through each transcript multiple times to capture the context and meaning. Initial analysis of early interviews informed the themes explored in interviews that followed. A codebook was developed independently by MNM, TFLM and AGW by reading through the transcripts and capturing the main, and sub-themes. The team then met virtually to compare and discuss their initial set of codes identified and generate an initial codebook. Data was manually coded by the research team, using an Excel spreadsheet, which involved employing language similar to that used by respondents, while particular attention was paid to the treatment and presentation of the themes. Main themes and sub-themes identified were implemented with agreed-upon definitions for each code to aid the coding process using a word document (Table 2).

We used a combined inductive and deductive coding approach that captured specific themes while leaving flexibility for new themes to emerge, particularly following the majority perspectives and experiences of participants. The dualistic technique of inductive and deductive thematic development analysis was informed by the work of Fereday and Muir-Cochrane which included the development and description of an analytical codebook [30]. Qualitative data from primary sources was analysed through content and thematic analysis. In this methodology, patterns, categories, and themes were organized from the bottom up by organizing data into ASRHR, teenage pregnancies and HIV testing, and COVID-19 context. This involved rigorous back-and-forth checks of the information to establish matching information. This approach was relevant in analysing the relationships between ASRHR, teenage pregnancies, HIV testing and COVID-19 responses, existing situation, and support provided to young people. The focus was placed on identifying, summarizing, and retaining data similarities, differences, and new emerging themes.

**Table 2:** Table of major and minor themes

Major Themes	Minor Themes
Priority shifts	Shift in focus and resources towards
	Compromised quality of service provision
	Limited AYP’s access to SRH information
Human Resource Challenges	Shortages of healthcare workers due to illness
	Reassignment
	Migration contributed to disruptions in service delivery
Supply Chain Disruptions	Supply chain disruptions
Compromised Service Quality	Decreased quality of SRH services
	Limited access to counseling and information.
Limited Access to SRH Information	School closures
	Restrictions on public gatherings limited AYP's access to SRH information and education
Suggestions for interventions to improve SRH services and lessons learned	Disaster preparedness strategy
	Use of community health workers and community-based ASRHR strategies
	Use of technology and social media platforms such as mhealth.

### Ethical considerations

Ethical and administrative approval was granted for all countries by local IRBs, the University of Malawi Research Ethics Committee (UNIMAREC), University of Zambia Biomedical Research Ethics Committee (UNZABREC) and National Health Research Authority, and the Medical Research Council of Zimbabwe (MRCZ). Due to the age of participants and the nature of the discussions, the research team set up several measures to ensure participants and parents fully understood the study requirements before agreeing to participate. First, assent was obtained from minors and written parental or caregiver consent was obtained from the guardians of the participants. Then interviews were conducted in a secure space to ensure the privacy of participants (only participants and researchers were present during interviews). Participants were also reminded of their right to withdraw from the study at any time, the right to decline to answer questions, as well as their right to choose to turn the audio recorder off at any time throughout the interview.

## Findings

We identified priority shifts as one of the main themes: how the emergency response limited access to SRH for AYP because of shortages of human resources, supply chain disruptions, compromised quality of service provision, and limited AYP’s access to SRH information. Another main theme included suggestions for interventions to improve SRH services and lessons learned: disaster preparedness strategy, increased funding for ASRHR, use of community health workers and community-based ASRHR strategies, and use of technology and social media platforms such as mHealth.

## Priority shifts: how the emergency response limited access to SRH for AYP

At the height of the pandemic, several stakeholders in Zambia, Malawi, and Zimbabwe reported that government and/or funding agencies had shifted both their focus and resources almost entirely to the response to COVID-19. This shift in focus and resources towards COVID-19 had a ripple effect on ASRHR and related programs, including outreach, education, and peer support. It resulted in reduced staff for routine services, decreased donor funding for SRHR, and diminished care for pregnant women, post-abortion patients and other sexual and reproductive health clients, who already faced limited resources before the pandemic. It was also reported that the provision of SRH services and outreach activities for AYP were commonly suspended or cancelled since, at the time, health facilities were overwhelmed with the COVID-19 pandemic response. In addition, outreach activities were either suspended or cancelled due to mandatory COVID-19-related restrictions. One key informant in Malawi described how the introduction of COVID-19 restrictions challenged how ASRHR activities were delivered.

COVID-19 caught the country by surprise. As a country, we did not have time to adjust. Restrictions made it even hard to adjust. The bigger challenge is that most of our activities are information-sharing activities that require gatherings. (KII Government Official MW)

In Zimbabwe, stakeholders reported that funds and capacity were diverted to COVID-19 response and to support the local healthcare system which had a depleting effect on SRH services. It was shared that most resources were shifted to the COVID-19 response including human resources.

And another issue that we also faced was prioritization, which was given to attending to COVID cases. This meant that the other services also suffered in the sense that they were not, especially when you look at the routine, reproductive, and health services. They suffered in the sense that people prioritized attending to cases of COVID-19. (KII Government Official ZW)

Some respondents also described how their project focusing on SRH needs of young people ended up asking for a reallocation of resources to accommodate the COVID-19 response. Additionally, in Malawi, Zambia, and Zimbabwe frontline healthcare workers or medical staff who were typically responsible for providing SRH services to young people were reassigned to other pandemic-related duties. Furthermore, spaces dedicated to providing SRH services to adolescents such as youth-friendly spaces were turned into COVID-19 testing points or waiting areas due to the limited space at the health facilities.

It was a little bit tricky because there are facilities, I will give an example of [redacted]. One of the facilities with a very active adolescent-friendly space had to give away the tent that they use for adolescent-friendly services to the COVID-19 case because there was nowhere else to put a patient if they tested positive. They would put that client in that tent for adolescent-friendly services. So, it was discouraging that even the peer educators had to temporarily be on hold, they were not coming to the facility to come and offer the services to their fellow peers. (IDI Facility staff ZM)

The case in Zimbabwe was similar, key informants discussed a lack of availability of SRH services for AYP. One stakeholder mentioned that at that time, organizations that supplemented government efforts to provide SRHR services to young people were closed indefinitely. The mere closure and the uncertainty that prevailed during the closure restricted access to services as there was no sure sign that service providers would resume.

It was really difficult because some of our clinics there were closed due to the unavailability of personal protective equipment [PPE], especially in rural areas. So, it was really difficult for young people to access services and I think due to the prioritization of COVID-19 patients, other issues that would come with young people to their facilities were now not considered as important. So sometimes you will be back home, simply because you don’t have the signs and symptoms of COVID. They were only concentrating on COVID-19 so it was a big challenge for us in terms of access to services. (KII NGO stakeholder, ZW)

Furthermore, stakeholders in Zimbabwe highlighted the critical role local businesses play in ASRHR by supplying young people with condoms. During the pandemic, many local businesses temporally closed or reduced their operating hours to reduce the spread of COVID-19.

It wasn’t just schools that were closed. Even some shops, you find that they close earlier, and some wouldn’t even open at times. So then with access to SRHR services like condoms and things like that, you’d find that you wouldn’t have them readily available as you would have before COVID, before lockdown and everything. So that was one of the factors that increased the teenage pregnancies and stuff. (FGD Adolescent, 18–24, ZW)

### Human resource challenges

Interview participants also reported that throughout the pandemic, there were shortages of already insufficient human resources, as the healthcare workforce was also infected with COVID-19. While it was reported that few staff died from COVID-19, others were placed in isolation centres which left a gap in service delivery. Notably, it was reported that these staff shortages in Zambia affected access to abortion, family planning or contraceptive, and HIV testing services.

There was something which could have made our teenagers not access some services because we had a high number of illnesses amongst the staff. The staff were getting sick because of COVID-19. So that could have affected the provision of some services to adolescents such as safe abortion. In some facilities which offer (abortion services), you would find that there is only one person who is trained in providing those things, and if that person is sick then it will affect the accessibility of that service. (IDI Facility staff ZM)

A similar situation was reported in Malawi, where one key informant explained that medical staff in their organization contracted COVID-19 which increased the workload on the remaining staff.

Some medical staff also contracted COVID-19 thereby increasing the workload for the remaining staff. At a certain time, two clinics were closed because more than half of the medical staff at the clinic tested positive for COVID-19.

(KII NGO stakeholder, MW)

Although brain drain occurred prior to the pandemic, it was severely worsened during the pandemic, especially since the pandemic hit mostly the higher income countries before most African countries. In Zimbabwe, a key informant mentioned that there was a noted brain and skills drain from Zimbabwe to other countries experiencing health workforce shortages, such as the UK, in consideration of the financial benefit of serving in the UK, compared to Zimbabwe. Thus, the lack of competitive remuneration for health workers contributed to the human resource shortages that were widely reported in the region.

Zimbabwe lost a lot of health workers, with some facilities losing their entire workforce; healthcare workers would rather go to the UK for caring as it made them a living against the rather poor remuneration offered to our national health workers. (KII NGO stakeholder, ZW)

### Shortages of commodities/supplies

The COVID-19 pandemic brought about several supply chain disruptions that lead to shortages of important SRH-related commodities and supplies which were still felt at the time of data collection. One stakeholder in Malawi explained that the supply chain for the commodities was disrupted and only COVID-19-related commodities were given priority. One stakeholder in Malawi described the scarcity of SRH commodities.

There were drastic periodic stock-outs of family planning commodities. These family planning commodities are procured centrally. It seems the priority for the government shifted from the procurement of family planning commodities to the procurement of COVID-19 response commodities. As an organization, we also started having problems with accessing condoms. It was believed that the companies that are major manufacturers of condoms went into mass manufacturing of gloves hence reducing manufacturing of condoms (KII NGO stakeholder, MW).

Both adolescents and stakeholders in Zambia indicated that there was a lack of supplies, including SRH commodities during the pandemic. In some cases, procurement of important commodities such as HIV testing kits and reagents was not done on time, leaving some health facilities with no commodities. A young person said,

It was very difficult to get tested or go to the health facility and get tested because of COVID-19. So, you would find that you go, they would want to test you quite all right, but they don’t have the instruments (supplies). (FGD, adolescent girls-14-18, ZM)

Adolescents and youth also reported the lack of essential medicines that addressed their health needs. For example, adolescents and youth not receiving treatment for STIs, information, or their requested supplies such as condoms. This shortage was overshadowed by the availability of COVID-19 supplies.

You would find that most medicine and the available vaccines were those for COVID-19. There was nothing to give for those with STIs (…) (FGD, adolescent boys-14-18, ZM).

### Compromised service delivery

The disruptions in service provision and commodity shortages also meant service quality was compromised. While it was policy to provide HIV counselling alongside testing services, some young people who had access to testing services were not provided with counselling. In Zimbabwe, it was mentioned that young people were viewed as a special group that needed counselling, and thus, the pandemic robbed them of this service, leaving them vulnerable to negative coping strategies after testing positive for HIV.

We have other ASRHR challenges that you want to discuss with these people, especially relationships about disclosure, etcetera. So, it was now that difficult for them to be equipped with the knowledge and skills, given confidence to cope with their HIV status. So, it really affected [us] a lot. (KII NGO stakeholder, ZW)

### Limited AYP’s access to SRH information

Disruptions in learning programs limited access to points where adolescents could access information. Although education and health policies mandate the provision of comprehensive sexuality education (CSE) to young people, it was revealed that during school closures and the implementation of distance learning, CSE was often dropped from the school curricula. In Zambia, it was reported that pre-pandemic, teachers provided information to adolescents weekly, to help them make informed decisions regarding their sexuality. However, the school closures led to adolescents and youth spending more time at home and without access to SRH information within the school setting.

We have been supporting the Tikambe (let’s talk) clubs but generally, they are called the “Anti-AIDS club” or the “AIDs Action Clubs. These are clubs where young people go to access information and share conversations or discussions, the challenges that they have, the peer-to-peer conversations that they have, or discussions on the challenges that they have regarding sexual reproductive or comprehensive sexual education. With the suspension of schools, there was no point where these adolescents could go and just ask for further information or if they wanted to ask for services or they wanted to seek clarity, or have a conversation. (KII ASRH program implementer ZM)

In Zimbabwe misinformation was a challenge, where young people turned to social media for SRH information which could have been misleading. There was no platform to ask questions or obtain further clarity on information received in the community.

So, the knowledge gap was created with COVID-19 through school closure. It really caused a lot of challenges among young people. Because even if you want to, you are told something in the community, there was no room for clarification. Sometimes you just use, those spaces that they have at school for comprehensive sexuality education, they no longer have access to that. (KII NGO stakeholder, ZW)

## Suggestions for interventions to improve SRH services and lessons learned

Throughout interviews, several stakeholders offered recommendations on how to maintain and modify SRH services during the pandemic to ensure that SRH information and services are accessible to adolescents.

### Disaster preparedness strategy

It was suggested by stakeholders in Malawi, Zambia, and Zimbabwe that on the organizational level, time and effort should be devoted to developing or updating a disaster preparedness strategy to ensure that future pandemics or other types of crises do not repeat the same types of debilitating effects. Stakeholders offered a range of suggestions for future disasters or crises such as establishing solid HIV self-testing protocols and stockpiling tests.

So, I think what’s, what’s important for now is to come up with a disaster preparedness strategy for us in Zimbabwe. So that even if we encounter another pandemic apart from COVID-19, we will be ready to face it because just like what I have said when COVID-19 came, every organization was closed in Zimbabwe. All operations they stopped because people didn’t know what to do next. So, this is really needed for us to make sure that even if we come to a pandemic, I will make sure that young people, they continue to have access to the services. (KII NGO stakeholder, ZW)

Furthermore, given that the COVID-19 pandemic tended to amplify certain weaknesses in the policy frameworks influencing ASRHR, it raised concerns regarding the age of consent for accessing SRH services. Some stakeholders discussed how critical improvements to specific policies are needed.

Our Public Health Act says that one with legal capacity should be able to access services, but in Zimbabwe legal capacity means that you’re 18 years old. So, it is our recommendation that the Public Health Act be reviewed to allow for those who are below 18 to be able to access services. So, this means that reviewing the age of consent to accessing services to ensure that adolescents who are in need of those services are prioritized and given their services. [This] will reduce, the high [levels of] teenage pregnancies, STI infections and new HIV infections that we continue to witness amongst the 10 to 24 years. (KII NGO stakeholder, ZW)*^1^

Moreover, the same stakeholder in Zimbabwe, also raised that more work could be done to elevate how all aspects of ASRHR should be prioritized as an essential service and specific safeguards need to be implemented to ensure that SRH services can be accessed. For example, restrictions on movement limited AYP access to SRH services during the pandemic.

It is important for the government to prioritize sexual reproductive health and rights as an essential service during pandemic, and that security forces need to be sensitized around sexual reproductive health and rights services. Because if you would go and tell a police officer that you had sex and want to go and buy contraceptive pills, you know, they would consider [this as] a minor issue, but the magnitude of that, you know, remains a big issue. So, it is important that the government should continue to prioritize sexual reproductive health and rights services. As a priority or essential service during pandemics and ensure that security services are sensitized around issues of SRHR. (Local NGO stakeholder, Zimbabwe)

### Use of community health workers and community-based ASRHR strategies

In all three countries, the important role that community-based volunteers play in terms of advancing ASRHR was highlighted. For example, in Zimbabwe, it was communicated that on the community level, throughout the pandemic, community members stepped up to keep programs running despite the challenges.

I know of the Stop the Bus campaign. Were uh, those mobile clinics were taken to adolescent localities. And they were offered the services. I think that was one of the interventions, which really helped young people to access services. But however, now the challenge is if the mobile clinic comes to your community, it means that if in your parents would also want, to access the service at the same facility. So sometimes it was a hindrance to us young people to access because you cannot stand in the same queue with your parents to access contraceptives. But it was really beneficial for other young people, those mobile clinics and all those campaigns that were being done at during this period. (KII NGO stakeholder, ZW)

It was also emphasized that given the current context of COVID-19, it is especially important to acknowledge the important roles that community members have in terms of providing SRHR information and care. In Zimbabwe, it was conveyed that this should also include the authentic engagement of young women and adolescent girls positioning them as local experts and community insiders. It was suggested that young women and adolescent girls should be invited to actively contribute to program development and adaptation through the sharing of expertise, community wisdom, and insights to identify and distil community-identified priorities and needs.

When we get into emergency situations, it can be very hard especially for our population to access services. But these community carers do have a very important role to play in terms of the provision of primary care to our communities, so I would want to recommend that we invest more and also rope in our adolescent girls and young women. They are not only recipients of services, but their active participation in terms of even evaluating the quality of services that are being provided to them [and] also contributing to giving recommendations in terms of how best we can improve in terms of service provision. … one of the lessons that we learned is when emergency it is mostly the most vulnerable that are affected the most. (KII Government Official ZW)

Service implementers lamented the need for guidelines to ensure the presence and sustained operation of spaces where adolescents can access information and services from community health workers, or through outreach services by health care providers, as they did during the pandemic.

A stakeholder in Malawi shared how they successfully trained young volunteers to deliver door-to-door services. Furthermore, stakeholders reported using community-based distributors of SRH services to increase access to ART services and contraceptives.

It’s good that in some communities there are some community-based distributors who are able to distribute certain family planning commodities to adolescents around and they are able to access them. So, I think that would be one good solution to provide these services, we could use community-based volunteers so that we minimize moving around or chances of transmitting that disease around. (IDI Facility Staff ZM)

### Use of technology and social media platforms such as WhatsApp (mHealth)

Throughout the interviews and FGDs, stakeholders acknowledged that the adoption of virtual platforms and technology was ‘eye-opening’ for their respective organizations. In many ways, at the organisational level, the utilization of technology (especially digital communication technology) improved and advanced communication flows allowing for a positive evolution during the COVID-19 period. This also provided a unique opportunity for organizations to reflect upon and reimagine how these tools can be utilized to improve organizational operations and potential service delivery. One of the strategies that organizations working with adolescents employed was to intensify social media engagement such as utilizing the use of platforms such as WhatsApp groups. Through these WhatsApp groups, they engaged experts who shared more information on particular topics. Adolescents were able to make clarifications when they needed more accurate information.

One of the strategies that we had employed in order to ensure that there is some continuity in accessing information…We intensified the creation of WhatsApp groups in each community. Where adolescents are from, the youth-friendly spaces where they joined, it became more like an online space via WhatsApp. Where the similar days they would meet at the facility, and they would then discuss issues to do with sexual reproductive health via those WhatsApp groups. So ideally the WhatsApp groups became the access points for adolescents to have conversations as well as signposting (KII NGO stakeholder, ZM).

Stakeholders in Zimbabwe also suggested similar strategies in terms of utilizing virtual and social media platforms to disseminate information to AYP on SRHR. For example, one organization described the use of WhatsApp, Facebook, and YouTube as tools that could be used to support information dissemination activities to facilitate more active engagement from populations of AYP.

There are examples of the young people who at some stage at university were part of the peer educators, who were running physical dialogues on a weekly basis where they discussed the different issues affecting them…Students simply just moved on and moved these [conversations] to the WhatsApp group, and that allowed us to then support them in terms of [continuing] with the discussions. In the same vein, we also went ahead and changed our support and ensured our online presence. We started generating a lot of content which we put up on our YouTube. We also started generating a lot of content, which we put on our Facebook pages and even on Twitter to allow young people to continue to access the information that they required. (KII NGO stakeholder, ZW)

In addition to the increased use of virtual platforms, stakeholders in Malawi and Zambia also described how during the pandemic, the radio was important for disseminating information about SRH. The radio is a useful tool that can transcend the urban and rural divide and thus reach both rural and remote populations and often more vulnerable populations.

## Discussion

Health and development problems including substance use, early marriages and alcohol consumption, STIs, HIV, early pregnancies, unwanted pregnancies, early marriages, and many more were exacerbated by the COVID-19 pandemic. While these challenges affected young people well before the onset of the pandemic, they worsened as governments around the world declared states of emergency- characterised by restrictions on or prohibitions of public gatherings, border crossings, and the availability and capacity of transportation services [31, 32]. The findings from this study highlight a myriad of challenges experienced by young people during the pandemic. Specifically, they add to the discussion on the direct and indirect influences of COVID-19-related restrictions on adolescents and young people’s SRHR. Sub-Saharan African countries experience the greatest gaps in the availability of essential adolescent healthcare services with many studies documenting the cultural and social barriers that prevent adolescents from accessing diverse services needed for their sexual and reproductive health [33, 34].

As health facilities shifted human resources and services towards the fight against the COVID-19 pandemic, the availability of youth-friendly services, spaces, and the adequacy of trained staff was compromised, as staff were repurposed to COVID-19 response services. Respondents revealed that youth-friendly spaces were turned into waiting areas for patients or used as isolation wards. This meant that services for young people provided in this space were no longer available. Though this finding is contrary to that of Kelly and Colleagues [35], whose study revealed that service providers continued to offer regular adolescent services that were available before the COVID-19 pandemic in South Africa, a study in Kenya, Uganda and Zambia had similar findings [13]. Furthermore, other studies also reported limited access to SRH services during the COVID-19 pandemic, as shown in this study [13, 14]. The COVID-19 pandemic affected the supply of contraceptive commodities- notably condoms, and disrupted the manufacturing and transportation of commodities, equipment, and the overall provision of SRH services [13, 36]. The study also noted that there were significant challenges in adolescent girls’ ability to access safe abortions due to restrictions and closures of primary health facilities. Similar studies have documented how such restrictions in service access create barriers for the survivors of sexual violence to equitably access healthcare resources needed to prevent unwanted pregnancies and infections [37].

Increased incidence of sexual abuse and unwanted intercourse, i.e., pressure to have sex without the partner’s consent during the pandemic’s peak period are reported elswhere [38, 39]. Health education to promote the usa of SRH care—including information, safe abortion, and contraceptive services for SGBV survivors was limited during the COVID-19 pandemic [36]. Further, the literature asserts that accessing services remains a challenge in rural areas because of long distances, issues associated with finding transportation, and associated costs [40]. Schools and community-based SRH programs provide girls with safe spaces, away from their perpetrators, and offer opportunities for guidance and counselling on how to avoid risky situations and behaviour. However, the unexpected closure of schools and drastic reductions in community-based SRH programs that support marginalized girls and protect young people from economic insecurity were seen as some of the major drivers of increased risk of teenage pregnancies. This was marked by reduced access to essential services, such as menstrual hygiene products, contraceptive methods, and other SRH services for adolescents and young people. Other studies highlighted how school closures increased the chances of school dropouts among young women [17].

Respondents indicated that community-based prevention services were disrupted by the pandemic, with the majority reporting that GBV prevention activities remained limited or unavailable entirely, similar findings to other community-based studies. These activities typically rely on face-to-face interaction or public gatherings and were either suspended or cancelled due to mandatory COVID-19-related restrictions in each context [41]. Furthermore, for ASRHR programming that builds community awareness and transformation over many years, such disruptions may result in the loss of prior gains or momentum. At the same time, this reality pushed organizations to develop innovative approaches to ASRHR programming. This was also seen in other countries in the East and Southern African region, for example, the Ugandan NGO Raising Voices recommended adapting SASA! community mobilization programming by moving discussions to virtual platforms such as WhatsApp, disseminating paper copies of activities for families, and using radio to air SASA! “Soap operas” [42]. Another innovation in the area of mHealth is the TuneMe mobile-based application developed by UNFPA which aims to equip adolescents with the information and motivation they need to make informed choices [43], even though not mentioned directly by respondents in this study, features of this App were widely used at the height of the pandemic to deliver SRH information to adolescents. Meanwhile, Hako and Shipalanga [44] described how radio, WhatsApp, recorded audios, and handouts were used to engage learners on SRH information when schools were closed during the COVID-19 pandemic in the Oluno circuit, Namibia.

In consideration of the experiences and perceptions of young people and other stakeholders, both in study contexts and from literature, we recommend renewed efforts to ensure a continued service provision for adolescents during pandemics, especially for those at risk of pregnancies, early marriage, and HIV infection to be treated as emergencies too. It would also prove worthwhile to recognize the different categories of adolescents and provide services that meet their specific needs during and after the onset of a pandemic. Sustained service provision can also be improved by leveraging community-based delivery systems for SRH services and the use of community health workers in the provision of services to young people. Further, the use of technological solutions such as mHealth may be appropriate to reach AYP with SRH needs. While GBV cases can be dealt with using multiple pathways (traditional and formal justice systems), integration of the traditional justice system into the mainstream justice system for GBV cases in the community during active pandemic periods and lockdown measures could be considered. Finally, in consideration of data and information systems, we recommend disaggregation of routinely collected SRH data by age, sex, and geography to understand gaps in service delivery to sub-populations during pandemics.

## Strengths and limitations

One of the main limitations faced by this study was the delays in obtaining ethical and administrative clearances. This component may have benefited from more attention if the initial timelines were maintained. Furthermore, there may also be some limitations regarding the focus on stakeholder organizations. Since the qualitative component of this study did not focus on the lived experiences of AYP, this is recommended as a natural next step for future research. However, it should be acknowledged that the team managed to conduct KIIs and FGDs with AYP in Zambia and Zimbabwe in the context of youth-led organizations. Although this limited representation, it was managed by the peer councillor’s availability to share their experiences. In addition, the study had sufficient variation from other stakeholders who have an interest in the health and well-being of AYP in the three study contexts, and this enriched the findings. Such representation also increased the transferability of study findings to other ESA country contexts— as they are characterized by similar sociocultural conditions.

Another limitation of the study involved data collection procedures since for the qualitative component some participants opted to be interviewed virtually. The research team provided each participant with the choice of either a virtual or in-person interview to facilitate convenience and ease of participation for organizational stakeholders. A further limitation was the 10 interviews from Malawi that were not audio recorded which resulted in missed verbatim transcription. The team acknowledges the various limitations that go with virtual data collection (connectivity issues, difficulty in reading nonverbal cues, or body language). Despite the liitations, the findings are useful for informing response in other global, regional and national emergencies.

## Conclusion

The COVID-19 pandemic brought with it a disruption of SRH services for AYP driven largely by pandemic related-restrictions, and diversion of resources/funding. Following this, there have been ripple effects on service delivery and access with potentially long-term consequences for AYP throughout the Southern African region. This underscores the need for maintaining and improving access to ASRHR services during future pandemics or other adverse events to meet AYP’s existing and emerging SRH demands. This study highlights how priority shifts, including a focus on pandemic response and resource allocation, compromised the quality of SRH service provision. Human resource challenges, such as healthcare worker shortages, reassignment, and migration, disrupted service delivery. Similarly, supply chain disruptions also contributed to limited access to essential SRH supplies, exacerbating the gaps in SRH service delivery. Further, school closures and restrictions on public gatherings significantly restricted AYP’s access to SRH information and education, leading to a decline in service quality and a decrease in access to critical services such as HIV testing, counselling, and contraception. The consequences of these disruptions are evident in the increased rates of teenage pregnancies, exploitation, child marriages, and gender-based violence (GBV) across Malawi, Zambia, and Zimbabwe. These findings highlight the need for targeted interventions, such as disaster preparedness strategies, the deployment of community health workers, and the use of technology and social media platforms (e.g., mHealth), to improve SRH service delivery and address the challenges faced by AYP during crises.

## Supplementary Information

Supplementary Material 1.

Supplementary Material 2.

## Data Availability

The data are not publicly available as it contains information that could compromise research participant privacy/consent. However, some anonymized aspects of the datasets may be available upon request and with permission of the UNFPA East and Southern African Regional Office. Note that data sharing is subject to UNFPA data sharing policies and data use agreements with the participating research centres.

## Abbreviations

ART: Antiretroviral treatment
ASRHR: Adolescent sexual and reproductive health rights
AYP: Adolescents and young people
FGD: Focus-group discussion
GBV: Gender-based violence
KII: Key-informant interview
NGO: Non-governmental organisation
SGBV: Sexual gender-based violence
SRH: Sexual and reproductive health
SRHR: Sexual and reproductive health rights
STI: Sexually transmitted infections

## Footnotes

1 Age of consent for accessing SRH services in Zimbabwe is 16 years old, while age of consent for marriage is 18 years old. These two ages of consent cause frequent contention, and impact adolescent access to SRH information and services (Public Health Act, Chap. 15:17, Zimbabwe 2018).
