# Supplementary material for: Exploring key challenges for healthcare providers and stakeholders in delivering adolescent sexual and reproductive health services and information during the COVID-19 pandemic in Malawi, Zambia and Zimbabwe: a qualitative study
Source: BMC Health Serv Res. 2024 Dec 4;24:1541. doi: 10.1186/s12913-024-11873-5 (PMC11616358; doi:10.1186/s12913-024-11873-5)
Supplement: Supplementary file 1 — Supplementary Material 1. [file 12913_2024_11873_MOESM1_ESM.docx]

Research Study on the Impact of COVID-19 on ASRHR, Teenage Pregnancies, and HIV

Testing

Interview Guide for Key Informant Interviews

**Section 1: Introduction**

1. Could you tell me about yourself? **Probes:**

1. What is your position/role in this organization?
2. What are your roles and responsibilities in this organization?
3. How long have you worked in this position?
4. What kind of work do you do that relates to adolescent sexual and reproductive health?

**Section 2: Situation of ASRH services**

2. Could you briefly tell me about adolescents’ and young people’s access to sexual and reproductive health services as well as on gender-based violence and child marriage in your country?

**Probes:**

1. Situation before COVID-19?
2. The situation during the COVID-19 pandemic?
3. Expected situation after COVID-19?

3. Could you tell me more about how school closures during the COVID-19 pandemic may have affected adolescents and young people in your country?

**Probes:**

1. Expected situation after COVID-19?
2. What youth-friendly health services that address SRH needs are available in your country?
3. How have they adapted when restrictive measures were in place?

4. In your opinion, could you tell us how adolescents and young people may have accessed SRH information during the pandemic?

**Probes:**

a. Was Comprehensive Sexuality Education available during school closures?

1. Could you briefly explain to me the impact of the COVID-19 pandemic and the accompanying country lockdown restrictions on the incidence of teenage pregnancies?
2. Could you briefly explain to me the impact of the COVID-19 pandemic and the accompanying country lockdown restrictions on the incidence of child marriages?

**Probes:**

1. The pandemic initiated both a hunger crisis and economic recession, globally. Could you tell us about how possible loss of income may have affected young girls?
2. In your opinion, how has the hunger crisis and economic recession affected SRHR for adolescents and young people?
3. What may be the longer-term effects that the COVID-19 pandemic will have in the future?

7. Could you briefly explain to me the impact of the COVID-19 pandemic and the accompanying country lockdown restrictions on the incidence of gender-based violence among young people and adolescents?

**Probes:**

1. How did the COVID-19 and related restrictions affect adolescent and young people’s access to social protection?
2. How did services and legal protection adapt to closures and shifting resources?

8. Could you briefly explain to me the impact of the COVID-19 pandemic and the accompanying country lockdown restrictions and access to reproductive health services?

**Probes:**

1. Was access to menstrual products affected?
2. Was access to safe abortion available throughout the pandemic?
3. Were there reductions in SRHR service provision?
4. Did SRH clinics/health facilities remain open?
5. Have you observed an increase in cases of teenage pregnancy due to the pandemic and accompanying measures?
6. Do you anticipate that such an increase will occur over the entire duration of the COVID-19 crisis and in the country as a whole?
7. Could you tell us about some specific interventions or adapted services that were adopted during the COVID-19 pandemic to ensure that adolescents and young people had access to SRH services such as HIV/STI testing, contraceptives, SRH information, and safe abortion?
8. Given the current context of COVID-19, what specific interventions, initiatives, or policy recommendations do you have to ensure adolescents and young people’s access to SRHR?

**Thank you for your time**

Note to interview: ask the participant to recommend other respondents working in adolescent sexual and reproductive health space
